# Effectiveness of non invasive external pelvic compression: a systematic review of the literature

**DOI:** 10.1186/s13049-016-0259-7

**Published:** 2016-05-18

**Authors:** Peyman Bakhshayesh, Tarek Boutefnouchet, Anna Tötterman

**Affiliations:** Department of Orthopaedics, Karolinska University Hospital, Karolinksa vägen, 17176 Solna, Stockholm Sweden; University Hospital Coventry and Warwickshire, Clifford bridge road, Coventry, CV2 2DX UK

**Keywords:** Pelvic fracture, Haemorrhage, Binder, External compression, Damage control

## Abstract

**Introduction:**

Pelvic fractures might carry a significant risk of bleeding. A wide variety of pelvic binders together with pelvic sheets are available and offer an adjunct to the initial management of poly-trauma patients with pelvic injuries. These devices are collectively referred to as pelvic circumferential compression devices (PCCDs). The aim of this study was to review the literature for evidence pertinent to the efficacy and safety of PCCDs.

**Methods:**

Using the PRISMA guidelines a systematic search on PubMed, Web of Science, CINAHL, Embase and Scopus was carried out. Articles included were in English language and published between 1999 and 2015. Studies included were appraised with narrative data synthesis.

**Results:**

Seven articles addressed mechanical properties of non-invasive external mechanical devices, six articles focused on physiological aspects, and three studies evaluated the pressure characteristics of these devices. We found 4 case reports regarding adverse effects. None of the studies identified addressed the cost effectiveness or pain relief issues related to the use of PCCDs.

**Conclusions:**

Based on available literature, PCCDs are widely used in the initial management of patients with suspected pelvic bleeding. There is evidence to suggest that external compression reduces disrupted pelvic rings. There are some complications reported following application of PCCDs. Hemorrhagic source and physiological effectiveness of PCCDs needs to be addressed in future studies. In the meantime judicious application of PCCDs will continue to be recommended.

## Background

The survival rate after pelvic fractures has improved during the recent years [1]. The overall high mortality has been shown to stem from concomitant injuries resulting from high-energy trauma rather than isolated pelvic fractures [1].

Fractures of the pelvic ring has been classified based on the vector of energy [2] to Lateral Compression types (LC) or Antero-posterior types(AP) or Vertical Shears (VS) and finally Combined Mechanism (CM). This fracture classification presented by Young-Burgess is commonly used in traumatology.

There is even other fracture classification system (Tile/AO) based on type of mechanical instability (A: stable, B: rotationally unstable, C: vertically and rotationally unstable) most of concern for pelvic surgeons [3].

Equally, temporary stabilization of pelvic ring fractures in patients with shock has been advocated by Advanced Trauma Life Support (ATLS) [4]. Historically, there have been many attempts to develop a treatment that will rapidly reduce and stabilize the disrupted pelvic ring in order to provide improved hemodynamic stability [5–7]. Until recently initial invasive surgeries such as the application of an external fixator [7] or a pelvic C-clamp [5] had been widely utilized. Such approaches were inadvertently associated with a time delay owing to the need for an operating theatre environment. In contrast, upon introduction of non-invasive external compression devices [8, 9], their application rapidly gained in popularity. Factors such as speed, safety, ease of application and their biomechanical capability to reduce a disrupted pelvic ring and pelvic volume have been advocated. External devices such as pelvic sheet or commercially available pelvic binders are jointly referred to as Pelvic Circumferential Compression Devices (PCCDs). There are different types of commercial PCCDs and the most widely employed brands are: Pelvic Binder® (Pelvic Binder Inc. Dallas, TX, USA), T-POD® (Bio Cybernetics International, La Verne, CA, USA) and SAM Sling® (SAM Medical Products, Newport, OR, USA).

Despite a relatively vast plethora of articles addressing the utilization of PCCDs, several questions remain unanswered. Are they able to minimize or stop the bleeding inside the bony pelvis? How long can external compression be safely applied over a disrupted pelvic ring? Are they effective in pain relief following pelvic trauma? Previous authors have only partly addressed such questions Spanjersberg et al. 2009 [10] and Cullinane et al. in 2011 [11]. With national consensus and management algorithms being introduced at the point of care, an up to date review of the evidence base is of the essence. Therefore, the aim of the present study was to systematically review the evidence for the efficacy of PCCDs in the management of hemorrhage following pelvic fractures. In addition, our review sought to evaluate the extent of complications and safety parameters associated with the use of pelvic compression devices.

## Materials and method

**Fig. 1:**
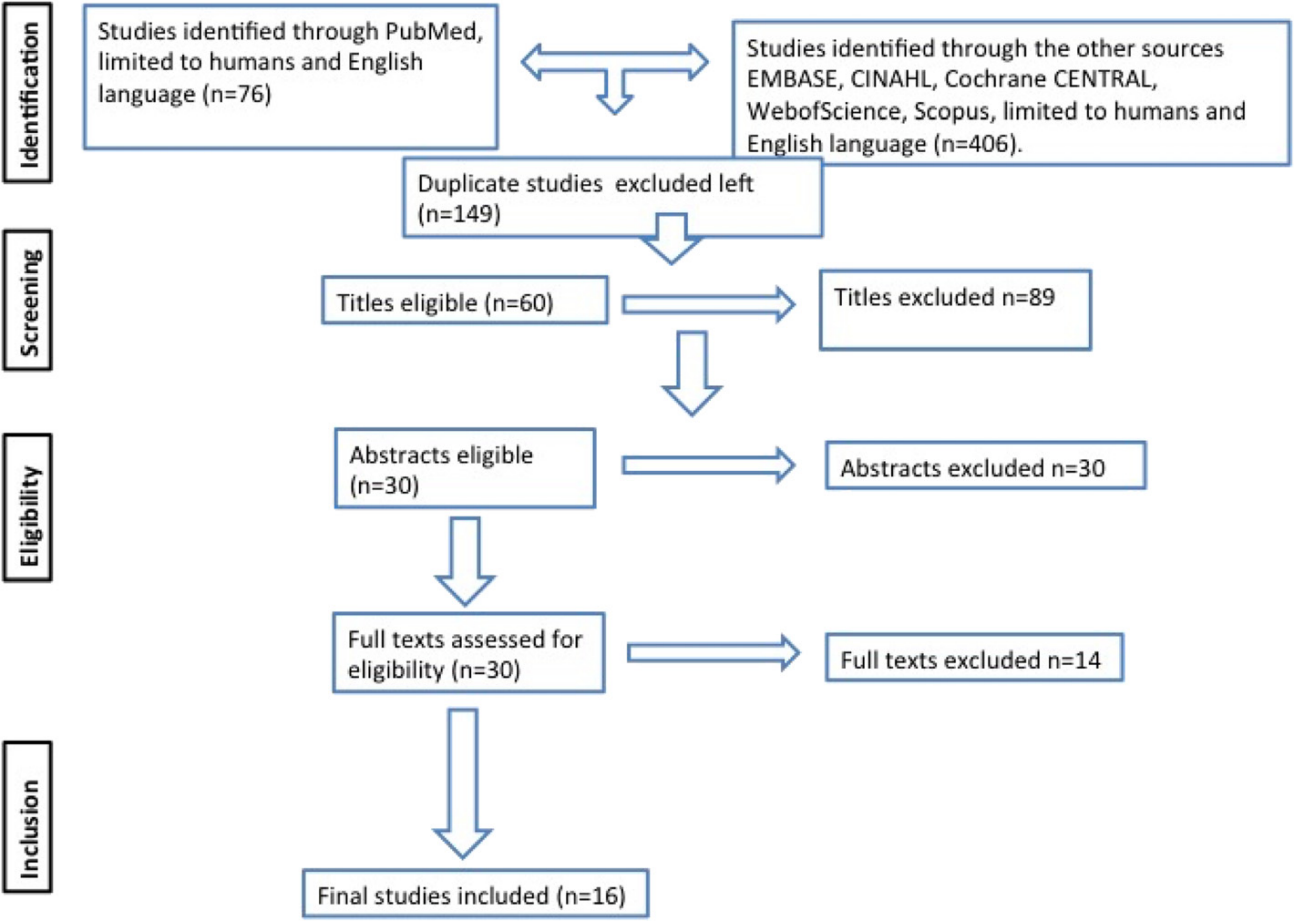
Screenings process

A systematic review of the literature was performed according to methods described in the Preferred Reporting Items for Systematic Reviews and Meta-Analyses (PRISMA) statement [12], this is outlined on the flowchart (Fig. 1). Studies included were original articles pertinent to our research questions. Therefore the inclusion criteria consisted of: Published studies in English language between the years 1999 and 2015 addressing non-invasive and temporary stabilization of pelvic fractures. Study designs consisting of randomized trials, case control studies; retrospective observational studies and case series were included. Case reports addressing efficacy of PCCDs were excluded due to their methodological limitations. Nevertheless, reference to these articles was included in the discussion in order to address the previously reported complications related to the use of PCCDs [13–16].

Exclusion criteria consisted of: External fixation devices, C-Clamp, surgical techniques, book chapters, and publication in other languages, as well as expert opinions, and commercial advertisements.

### The search strategy followed a standardized approach

Medical Subject Headings (MeSH) terms were used with the search string: “Pelvic” and “Fracture” and (“Binder” or “T-POD” or”Sheet” or “Wrap” or “Temporary Stabilization” or “PCCD” or “Sling”). These terms were sequentially searched within the most commonly used biomedical indexing databases: Pubmed, CINAHL, WebOfScience, Scopus, Cochrane Library and Embase. Databases were accessed through Karolinska Institute in Stockholm, Sweden.

Articles, which met the inclusion criteria, were systematically assessed for the relevance of their content (Fig. 1: depicts a flow diagram of the systematic literature search). Initially titles were screened for primary inclusion and exclusion. All the abstracts obtained were further assessed for eligibility. The full texts of articles, which met the relevance and inclusion criteria, were obtained and reviewed, paying particular attention to relevance to our research questions. Access to full text articles was obtained from Athens (Eduserv©) and Karolinska university library. A rigorous systematic search was performed using the criteria outlined above. References were transferred into Endnote referencing software® (Thomson Reuters) and duplicates were discarded. Firstly titles and abstracts were reviewed for relevance according to the research question. The remaining studies were analysed in their entirety. References of full texts were also reviewed to identify any other potentially relevant studies. The acquisition of articles is summarised in the flow diagram (Fig. 1). The final studies were reviewed according to study design, analysis and interpretation as well as validity of results. Two independent reviewers have critically appraised (PB and TB). Where there was discrepancy, an agreement was reached by consensus. The critical appraisal followed a systematic approach guided by the consolidated standards of reporting trials (CONSORT) for randomised studies [17], and the validated Methodological Index for Non-Randomised Studies (MINORS) [18]. A comprehensive critical appraisal checklist was created and used alongside the final papers included in the review (Table 1).

**Table 1:** Results

Authors, year	Study design	Pressure Characteristic	Mechanical aspect	Physiological aspect	Level of evidence	Control group	Duration	Number of cases	Results clinically applicable	Critical comments
Bottlang 2002	CS	+	+	-	V	0	?	7	+	Level of greater trochanters
Krieg 2005	CoS	-	+	-	III	0	16 m	13	+	Early physiological response
Croce 2007	RS	-	-	+	IV	+	10y	186	+	Reduced transfusion rate and hospital stay
Ghaemmaghami 2007	RS	-	-	+	IV	+	4y	237	+	No effect of PCCD
Jowett 2007	CS	+	-	-	V	-	-	10	+	Risk for pressure sore
Nunn 2007	CS	-	-	+	V	-	?	7	+?	Early physiological response
DeAngelis 2008	CS	-	+	-	V	+	-	10	+	Cadaveric. Mechanical effect.
Tan 2010	CoS	-	+	+	III	_	4y	15	+?	Early physiological response. Good mechanical effects
Knops 2011	CS	-	+	-	V	+	-	16	+	Cadaveric. Mechanical aspects
Knops 2011	RCT	+	-	-	II	+	-	80	+	Healthy adults
Knops	Lab CS	+	-	-	V	-	-	-	+	Useful model, pressure effects
Prasarn 2012	CS	-	+	-	V	-	-	5	+	Cadaveric, mechanical effect
Pizanis 2013	CoS	-	-	+	III	-	8y	192	+	Open cohorts register study, No control
Prasarn 2013	CS	-	+	-	V	-	-	9	+	Cadaveric, mechanical
Prasarn 2013	CS	-	+	-	V	-	-	5	+	Cadaveric, mechanical
Fu 2013	RS	-	-	+	IV	+	4y	585	+	One centre, retrospective data

*CS* Case Series, *CoS* Cohort Study, *RS* Retrospective Study, *RCT* Randomized Controlled Trial

## Results

Following application of the eligibility criteria a total of 16 studies were identified. Seven articles addressed the mechanical properties of non-invasive external mechanical devices, six articles reported on the physiological aspects, and finally three studies evaluated the pressure characteristics of these devices. None of the selected studies had addressed the cost effectiveness issue related to the use of PCCDs. Similarly, none of the studies employed immediate effect on pain relief as an outcome measure. Adverse effects of external pelvic compression were found to be scarcely reported with four case report articles [13–16]. Due to marked heterogeneity amidst all the studies, it was not possible to combine the results for meta-analysis of the reports presented.

## Mechanical effects

Bottlang [19] et al. 2002, in a cadaveric study consisting of 7 human cadavers induced 2 patterns of fractures B1 and C1 (Tile, OTA classification). Satisfactory reduction at the level of symphysis pubis and posterior aspect of sacroiliac joints was achieved with application of a PCCD. Level of greater trochanters was shown to be the level of choice for the generation of a force reaching 177 ± 44 N for B1 types and 180 ± 50 N for C1 types. An intra-peritoneal pressure of 6.2 ± 5.8 mmHg was reported and a local pressure of 24mHg, with PCCD application at the level of greater trochanters.

Krieg et al. [16] in a prospective cohort study monitored the mechanical characteristics of PCCD on 13 patients with pelvic ring injuries. Their results demonstrated fracture-reduction properties of PCCD when applied on external rotation type fractures, comparable to that of internal fixation. The last authors were unable to identify any significant over reduction in internal rotation type fractures either. In this study no significant complications had been reported apart from one case with minimal skin abrasion. The authors recommended intermittent monitoring of patient’s skin under PCCDs.

DeAngelis et al. [20] in a cadaveric study reports the superiority of T-POD when compared to pelvic sheet wrapping in reduction of diastasis at the symphysis. No physiological aspects have been included in this study. Similarly, these authors did not define the pressure characteristics associated with T-POD and required to maintain the reduction.

Prasarn et al. [21] compared external fixator with T-POD in five cadaveric specimens in the context of simulated Tile-AO type C injury and electromagnetic motion device. Extent of movement was measured in simulated logrolling, bed transfer and head-up tilt. Although not statistically significant superior results have been demonstrated with T-POD compared to external fixator. In this lab based controlled environment it was not possible therefore to directly extrapolate findings onto clinical practice. The same authors group conducted further analysis comparing the position of T-POD application at the level of the greater trochanters and the anterior superior iliac spine. In this yet another simulated unstable cadaveric fractures, motion analysis demonstrated better stability in sagittal, coronal and axial planes when the T-POD is applied over the greater trochanters as per manufacturers recommendations [22].

Circumferential pelvic bed sheet was compared to T-POD sling in the context of simulated cadaveric rotationally and vertically unstable pelvic fractures. In a similar approach to the previous studies, motion analysis was performed during application of the device, logrolling, bed transfer and elevation of head end of the bed. The results demonstrated no differences in stability conferred by either device during application or motion [23].

Knops et al. [24] compared the results of three different commercially available PCCDs in terms of stability achieved using simulated cadaveric pelvic fractures. Authors demonstrated effective reduction and stability of all three types (T-POD, SAM-sling and Pelvic Binder) to reduce all types of pelvic fractures. No undesirable over reduction was reported and the T-POD was found to require the least amount of pressure and pull force to achieve a similar degree of reduction and stability when compared to the other two devices.

## Pressure characteristics

**Fig. 2:**
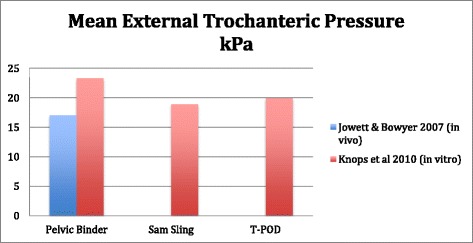
Crude mortality rates^★^ associated with lethal pelvic bleeding and the application of PCCDs reported by three different studies

Knops et al. compared three different types of PCCDs (T-POD, SAM-sling, and Pelvic Binder). All three showed pressure characteristics measured around the bony prominences to be above 9, 3 kPa (See Fig. 2). The highest pressures were noted while individuals were placed on a spinal board. Such pressures have been shown to increase the risk of skin sores when applied for a longer period [25]. Based on these studies early transfer to a hospital bed and early removal of the binder was recommended. Similar superficial pressures were recorded at the level of greater trochanter and sacrum by other groups both in healthy individuals [26] and simulated cadaveric studies [27].

## Physiological effects

**Fig. 3:**
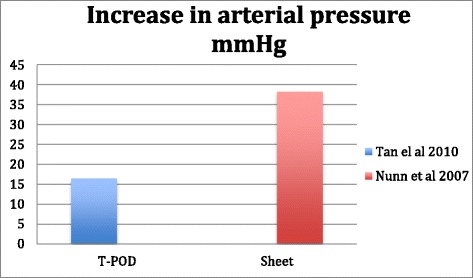
Transfusion requirements in mean unit packed red cells^❖^ within the initial 24 h associated with the application of PCCDs reported by four different studies

Tan et al. 2010 [28] conducted a prospective cohort study of 15 patients with pelvic fractures. They monitored physiological and radiological effects of PCCDs. This cohort study shows clear response of pulse 106 ± 6.8 vs. 93 ± 4.9, the mean arterial pressure (MAP) 64.7 ± 6.4 vs. 81.2 ± 6.4 before and two minutes respectively following application of PCCD on a disrupted pelvic ring. Pelvic radiographs were obtained before and directly after application of a pelvic sling within five minutes. The level of greater trochanters was determined as the optimal level of application. In this study factors such as: age, mechanism of injury, injury severity score, time from injury to, fluid resuscitation and number of concomitant injuries were taken into consideration. Adjusting for these multiple confounders showed that external compression had statistically significant effect on heart rate and symphysis diastasis. MAP (See Fig. 3) remained significant in backward correction (*p* = 0.027) but not with forward correction (*p* = 0.078).

**Fig. 4:**
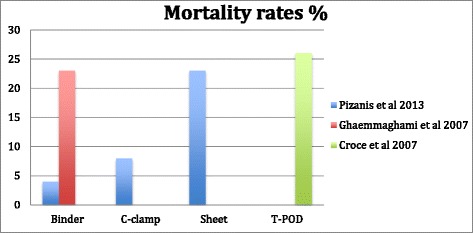
Results of pressure analysis in mean external trochanteric pressures (kPa) associated with different PCCDs reported by two different studies

**Fig. 5:**
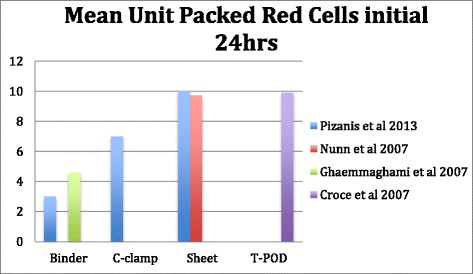
Mean change in arterial pressure (mmHg) associated with the application of T-POD and Pelvic Sheet. Independent figures reported by two different studies

Pizanis et al. [29] 2013, in a retrospective analysis of prospectively collected data obtained from the German Pelvic Trauma Registry, compared binder (*n* = 28), pelvic sheet (*n* = 31) and C-clamp (*n* = 133). Results of this analysis indicated a higher percentage of fatal bleeding based on clinical, radiological and autopsy results in the pelvic sheet group compared to C-clamp and binder group (23 % vs. 8 % vs. 4 %)(See Figs. 4 & 5). However in this study patients in the binder group were younger compared to c-clamp and sheet group (median age 26 vs. 42 and 47, *p* = 0.01). The authors have pointed out that patients who received a sheet wrapping may have been treated in centers less equipped to receive major trauma. A major limitation of the study has been missing data regarding adequate application of these devices.

Croce et al. 2007 [30], in a 10 years retrospective registry analysis compared the results of invasive external pelvic fixators vs. PCCDs. The inclusion criteria in their study were multiple pelvic ring fractures associated with vascular disruption and severe retroperitoneal hematoma, open book fractures (APC II, III). Total number of patients was 186 admitted to Trauma Center in Memphis. Each group had 93 patients who either received external pelvic fixator or PCCD. In the PCCD group 24 h transfusion (4,9 vs. 17,1 U *p* < 0.0001, see Fig. 3), 48 h transfusion (6 vs. 18.6 *p* < 0.0001) were statistically lower than externally fixed group. Hospital length of stay in PCCD group (16.5 vs. 24.4 *p* < 0.03) was shorter. Mortality (See Fig. 4) was also shown to be lower in PCCD group but not statistically significant (*p* = 0.11). Patients in external fixation group had higher mean ISS (33.6 VS. 38.6 *P* = 0.02). During the 10-year study period a decrease in the overall utilization of initial external fixators was noted.

Ghaemmaghami et al. 2007 [31], in a retrospective study between 2002–2006 compared patients with PCCD (118 patients) with patients without PCCD (119 patients). Inclusion criteria in their study was 1) Fracture type APCII, III and LC II,III or Vertical Shear 2) Patients older than 55 with pelvic fracture 3) Pelvic fractures and Systolic Blood pressure under 90 mmHg. In this study the authors found no benefit of PCCD regarding mortality rate (See Fig. 4), need for angioembolization or 24 h transfusion (See Fig. 5). However in this study all type of fractures have been included, and particularly in PCCD group it has been more stable type of fractures (LC1 24 % in PCCD group vs. 8 % LC2 12 % vs. 8 %, LC 3 12 % vs. 17 %). The authors concluded that a subgroup of these patients could benefit more from the application of a PCCD. Nevertheless, their results were not significant enough to support this observation.

Fu et al. 2013 [32] in a retrospective study compared two groups of patients, those who received a PCCD upon arrival to a trauma center, with those who did not. During 53 months, 585 patients who were transferred to a major trauma center in Taiwan had been included. The patients with unstable pelvic fractures who received PCCD had fewer transfusions (398.4 ± 417.6 ml vs. 1954.5 ± 249 ml *p* < 0.001) shorter intensive care duration (6.6 ± 5.2 vs.11.8 ± 7.7 *p* = 0.024), shorter hospital stay (9.4 ± 7 days vs.19.5 ± 13.7 *p* = 0.006). They even show better results in patients with stable types of fractures, who received PCCD while considering transfusion requirements, intensive care length of stay and hospital length of stay. The authors have mentioned restrictions of this study as low number of patients, retrospective nature of study and limitation of being performed in one center. The sample size is much larger than previous studies.

Nunn [33] et al. in 2007 reports the physiological outcome of pelvic sheet wrapping on pelvic fractures. In a series of seven patients they introduce a technique of internal rotation of the thighs and binding of legs and thighs, then sheet wrapping of pelvic ring. In this series they report one death, which is reported because of brain injury. They had one LC III –type fracture in this series. Authors report a dramatic change in blood pressure and pulse 15 min post application of improvised pelvic binder (See Fig. 3). In this series almost all the patients needed substantial amounts of blood and fluid resuscitation, which has even been advocated by the authors. The spontaneous increase in the blood pressure might be interpreted as a transient phenomenon. The authors highlighted the need for a post reduction X-ray to verify the quality of reduction.

## Adverse effects

We found 4 case reports regarding complications with PCCDs. Complications such as skin break down; catastrophic myonecrosis and bilateral peroneal nerve palsy have been reported [14–16, 34]. Schaller et al. reported an isolated case of skin breakdown following the use of circumferential pelvic sheet; this complication precluded subsequent definitive internal fixation surgery [12]. In contrast Shank et al. reported a case of bilateral peroneal nerves neurapraxia following the use of external compressive wraps. The latter was limited to an isolated case and it was unclear of this complication was due to the technique of application or other predisposing factors [13].

## Discussion

All the studies included in the systematic review suffered from combined and repeated methodological deficiencies. There was a lack of clear statement indicating the primary objective of the study and whether a comparison of the efficacy of treatment methods was the primary endpoint. Exclusion of patients who received treatment but did not have their outcomes reported were not clearly outlined. These methodological limitations also included a lack of prospective calculation of sample size, power and level of significance; and no evidence of study registration or conduct according to a predefined and registered study protocols. Due to the lack of intransigent evidence supporting one or more PCCDs model there was no scope for adequate standard control comparison groups. In addition, there was no evidence of blind assessment of objective study endpoints with lack of statements justifying the absence of blinding. This systematic review of the literature highlights the paucity of high quality controlled studies investigating the efficacy of PCCDs. Such literature remains poor with heterogeneous cases and predominantly relying on simulated environment.

Currently there are no international consensuses, apart from recommendations regarding application of PCCDs [4, 11, 35, 36].

Pre-hospital pelvic examination of pelvic fractures has shown low sensitivity 59 % [37]. Some authors have advocated broader application of PCCDs [38].

Based on published literature [28, 33] there is evidence to believe that application of PCCDs gives raises in Mean Arterial Pressure. However despite lack of current evidence one might ask if this raise is in favour of poly-trauma patients while the source of bleeding has not been addressed and managed.

While retroperitoneal space has been reported to be an open space [39] there would be a hypothetical fear to squeeze the blood out of the pelvis or the ruptured vessels into retroperitoneal space. This fear might be more relevant in LC-type fractures or in Complex type fractures with injuries to acetabulum and quadrilateral plate.

However we could not find any evidence to confirm this hypothetical fear.

Pre-hospital application of PCCDs mandates prompt educations for medics on the field. Even if there are no reported data that application of PCCD on LC-type fractures can be hazardous but “No More Harm” principal of ATLS must be considered [4]. Published literature shows a need for further education upon application of PCCDs [40, 41].

On the other hand if we have considered that PCCDs are an adjunct during primary resuscitation, their application might be considered as soon as possible [38].

The cost-benefit study of this type of approach has not been published.

There are published data highlighting lack of training and knowledge regarding indications and proper application of PCCDs [40, 41].

PCCDs seem effective to reduce a pelvic ring fracture, however this reduction might reasonably be considered for AP-type of injuries in haemodynamically unstable patients [19–23]. Literature is in favour of PCCDs physiological effect during early phase of resuscitation [28, 33]. PCCDs overall outcome, regarding mortality, hospital length of stay, ICU length of stay etc. is controversial [30, 31]. PCCDs might be a feasible option upon transfer of the patients to trauma centres [32].

Based on published studies almost all type of PCCDs induces a pressure effect over bony prominences around pelvis higher than 9,3kpa [24, 26, 27]. This can theoretically induce pressure ulcers after 2–3 h [25]. This makes PCCDs as a non-feasible option as a bridging to definitive fixation. However some authors recommend an early internal fixation [42, 43] while the others recommend damage control approach [44] or staged approach [45].

PCCDs have shown superior mechanical results compared to External fixation [21] which might be because of their circumferential pressure in the presence of anterior and posterior disruptions [21].

Recent pelvic volume studies [46] have criticized the past believes [7] and showed that volume changes in true pelvis are not that dramatic that previously believed.

PCCDs can obviously not stop an arterial bleeding, Grimm et al. [39]. Bearing in mind that the retroperitoneal space is not a closed compartment [39] raises the issue that blood can be pushed to this area while application a PCCD on any type of pelvic fractures. However, the study by Grimm has been done on cadavers, and water has been used instead of blood. Both of these factors could have had an effect on the results. Further studies are needed in the future to better outline this point.

The initial rise in MAP and fall of pulse rate, which have been shown, might be a transient effect, and there are no data to believe that these effects would be persistent over time [28, 33]. The authors have properly engaged other measures in the resuscitation of these critically ill patients, which adds effect of cofounders to the results. Even in this subject there is a need for further studies in the future.

Considering Mortality, Mean Unit Packed Red Cells Transfusion, increase in Mean Arterial Pressure and Mean External Trochanteric pressure as outcomes have been summarised in our figures (Figs. 2, 3, 4 and 5).

In Fig. 4 patients in Pizanis et al. and Croce et al. studies [29, 30] had both higher ISS (35 and 36) compared to study by Ghaemmaghami et al. [31] there mean ISS were around 22,6. Croce et al. had higher mortality in their material while using T-pod however this mortality rate was even higher in External fixation group [30].

## Conclusion

Mechanical aspects of PCCDs have been studied well. PCCDs have shown promising results to reduce a disrupted pelvic ring. Short-term physiological effects of PCCDs on blood pressure and increase in MAP have been shown in case series. Long-term physiological effect on outcome, regarding mortality, hospital length of stay and transfusion rate etc. is controversial and needs further studies. Cost-benefit studies needs to be performed to justify broader applications of PCCDs in pre-hospital setting. Potential adverse effects of PCCDs in terms of pressure effects on soft tissues needs to be further addressed and weighed against potential benefits.

Low-pressure venous bleedings and bleeding from the fracture sites are self-tapping in the absence of coagulopathy. When coagulopathy is threatening application of binder in certain type of fractures (AP-types) might be justified until coagulation goals during initial resuscitation phase have been reached. Trauma services might consider registering times for application and removal of PCCDs and proper training for application of PCCDs. These implementations make future studies much easier.

Awareness needs to be raised regarding PCCDs limitation to stop arterial bleedings.

Intermittent check of skin under PCCDs and avoidance of prolonged application of PCCDs is recommended.

Consequently, the current body of evidence cannot be used to provide clear recommendations and further research is required to define clinical, mechanical and haemostatic outcome measures. Utilising the latter, one might conclude that priority should be given to randomized controlled trials. Only robust long-term results can truly support externally valid recommendations on the utilization of pelvic circumferential compression devices. However, these types of studies in critically ill trauma victims raise ethical issues.
